# Mannerisms and stereotypies in catatonia: beyond simple motor movements

**DOI:** 10.3389/fpsyt.2024.1435719

**Published:** 2024-09-12

**Authors:** Yassir Mahgoub, Aum Pathare, Dallas Hamlin, Hailey Kindt, Andrew Francis

**Affiliations:** ^1^ Department of Psychiatry and Behavioral Health, College of Medicine, The Pennsylvania State University, Hershey, PA, United States; ^2^ College of Medicine, The Pennsylvania State University, Hershey, PA, United States

**Keywords:** catatonia, mannerisms, stereotypies, descriptive psychopathology, DSM, autism spectrum disorder DSM: diagnostic and statistical manual

## Abstract

**Background:**

Catatonia is a complex syndrome with prominent psychomotor, cognitive, and affective manifestations. Among the commonly described manifestations of catatonia are mannerisms and stereotypies. Kahlbaum, who coined the term catatonia, described several presentations of mannerisms and stereotypies as complex behaviors in his monograph. However, most of the subsequent psychiatric literature has described both phenomena in the context of simple motor movements or actions.

**Study design or method:**

We identified complex behavioral presentations of mannerisms and stereotypies described by Kahlbaum in his monograph. We summarize the development and use of mannerisms and stereotypies as psychiatric terminology since Kahlbaum, emphasizing the spectrum of behavior captured early in this usage. We list the inconsistent and interchangeable use of these terms in subsequent literature and describe recent examples of complex behavioral manifestations of mannerisms and stereotypies in the context of catatonia.

**Study results:**

We propose a new framework for mannerisms and stereotypies that utilizes descriptive psychopathology factors in various normative references, the context of the behavior examined, and critical pathological processes identified in mannerisms and stereotypies to identify and describe complex manifestations of these phenomena.

**Conclusion:**

Catatonia continues to remain under-recognized and under-treated. Our current diagnostic tools can make mannerisms and stereotypies complex and challenging to recognize. We suggest defining stereotypies as non-contextual repetitive activities while mannerisms as non-contextual peculiarities of activities. Utilizing our proposed framework and definitions can improve the description, recognition, and treatment of catatonia.

## 1 Introduction

Catatonia is a complex syndrome with prominent psychomotor, cognitive, and affective manifestations that occur in association with several psychiatric and medical conditions (1). Karl Ludwig Kahlbaum coined the term catatonia and described the course of 26 patients with catatonia, highlighting several varieties of presentation (2). Catatonia continues to remain significantly underrecognized and undertreated. Several catatonia presentations were described in psychiatric literature, including mannerisms and stereotypies. In the context of catatonia, the DSM-5-TR defines mannerisms as odd, circumstantial caricatures of normal actions, while it defines stereotypy as repetitive, abnormal, frequent, non-goal-directed movements (3). The psychiatric literature has provided examples of both mannerisms and stereotypies limited to simple movements or actions, ignoring the complexity of presentations of catatonia as complex behavior. This limitation resulted in the poor recognition and treatment of some complex manifestations, such as polydipsia and excessive eating (4–6).

Several factors challenge the recognition and description of complex manifestations of mannerisms and stereotypies. The extant literature reveals inconsistent and occasionally interchangeable descriptions of mannerisms and stereotypies. DSM-5-TR descriptions and definitions vary depending on the disorder under which they are subsumed, such as catatonia, autism spectrum disorder (ASD), and stereotypic movement disorders, in addition to a separate definition in the appendix section (3). Moreover, using terms such as odd, goal-directed, or non-goal-directed creates ambiguity that needs to be clarified. Finally, recognizing the complex manifestation of mannerisms and stereotypies requires an in-depth understanding of various norms and contexts used to assess and qualify.

## 2 Methods

We identified complex behavioral presentations of mannerisms and stereotypies described by Kahlbaum in his monograph. We used the English translation of the Kahlbaum monograph, translated by Y. Levij, M.D., and T. Pridan, M.D., translated in 1973. We summarized the development and use of mannerisms and stereotypies as psychiatric terminology since Kahlbaum, emphasizing Kahlbaum’s spectrum of behavior captured early in this usage. We listed the inconsistent and interchangeable use of these terms in subsequent literature and describe recent examples of complex behavioral manifestations of mannerisms and stereotypies in the context of catatonia.

## 3 Results

### 3.1 Kahlbaum’s description of repetitive fixated or peculiar motor and behavioral manifestations of catatonia

Kahlbaum’s 1874 monograph notes several presentations of catatonia, including mutism, immobility, stupor, staring, posturing, echo phenomena, stereotypies, mannerisms, grimacing, verbigeration, rigidity, negativism, perseveration, withdrawal, waxy flexibility, catalepsy, combativeness, and excitation (2). His description includes behaviors ranging from simple to complex and highlights the overarching phenomena and the discrete symptoms. An example of this is the cluster of entities that would now be subsumed under grimacing, including spastic contractions of the facial and cervical muscles (cases 1,5), prolonged tremors of the eyelid muscles (case 1), convulsive cramps of the jaw (case 3), peculiarities of the mouth movements and snout-like expressions (case 6), cramps of the temporal, masticatory muscles and eyes (case 10), and gnashing teeth (case 17).

Similarly, Kahlbaum’s monograph used terms like convulsions, epileptiform attacks, tremors, cramps/tetanic cramps, and spastic contractions, sometimes interchangeably, to characterize contractions of different muscle groups in a way that resembles his descriptions of facial movements (2).

Kahlbaum also used stereotypy, stereotypical behaviors, idiosyncrasies, and compulsions to describe non-contextual patterns of repetitive or peculiar behaviors. As we describe some of these vignettes, we will assign these complex behaviors into either mannerisms or stereotypies based on our proposed definition described in section 4.3 Some of these presentations were not explicitly characterized as stereotypical, as in case 3, where catatonia was noted in a 45-year-old female who developed depression alternating with mania. This vignette describes a patient who engaged in a series of non-contextual repeated activities ranging from pathological obsessions with cold water to repeated twisting of the garment and clothing in a sausage shape (stereotypy). Kahlbaum notes,

“*The disorder was associated with very obvious idiosyncrasies, strange behavior, and frequently occurring states of nervous excitation … she decided with tremendous conviction that the only treatment that would help her was a cold-water cure; the same winter, she underwent a rigorous cold-water cure on an intensive scale. She thus acquired physical tolerance to cold and, after this course, remained a fanatic adherent of cold-water cures to an extent that bordered on pathological obsessions………She sat quietly on the couch, constantly twisting a garment in her hands, and gave curt answers to questions put to her. This habit of twisting a piece of clothing into a sausage shape had become such a stereotyped activity that the female orderlies attending to her appropriately called it “sausage making*”.

Additionally, Kahlbaum described repetitive head rubbing and undressing in case 4 (stereotypy), a patient with mania and catatonia. In this case, he reported,

“*This was followed by apathy, during which she was obsessed with rubbing all hair off her head.*”

He later described her progress:

“A state of apathy prevailed, and she showed an obsessive need to undress”.

In case 6, Kahlbaum described the course of a 24-year-old male who presented with catatonia. He described a non-contextual fixated repetitive pattern of a patient eating directly from the plate without using cutlery or fingers associated with other catatonic features (stereotypy). He stated,

“*Here at home, he ate without using a knife or spoon but insisted stubbornly on drinking soup from the plate; even when eating meat, he was prone to avoid using his fingers. He showed striking peculiarities of body posture at rest, particularly during movements when he could be induced to be brought out of his passive behavior*”.

Case 14 described a 22-year-old male who presented with a manic episode that alternated with depression. He described a pattern of non-contextual fixated peculiarities of presentation, such as exaggerated politeness (mannerism), in addition to non-contextual fixated repeated activities, such as repeated kissing hands (stereotypy) in the context of catatonia. He reported:

“*During the period of melancholic agitation, he was reluctant to be alone. He appeared anxious and showed a cringing politeness toward the physicians and others. For example, he persisted in kissing their hands, coats, or even their feet, and only once did he behave in a markedly polite way. He complained readily and in an exaggerated manner when he had some pain due to minor intercurrent disease……He showed all kinds of singularities in regard to his clothes and other aspects of his external appearance, and in all these abnormalities, the most prominent phenomenon was the persistent repetition. For example, he often kissed the hands of his physicians again and again*”.

Other presentations of complex, non-contextual, fixed, repetitive, or peculiar patterns of behaviors were described in cases without characterization, including a presentation from case 2, a 33-year-old male with depression who had displayed mannerisms in her breathing and her pattern of leaving food. He reported:

“*During examination by a physician, there was no change in his behavior: his eyes could be opened only by force, and he could not be induced to utter a single word. His facial expression was melancholy and anguished; when at rest, his breathing was slow and regular, but it quickened and became somewhat uneven with a sighing quality during medical examination……he also had the peculiar habit of leaving some food even if given minimal quantities*”.

Case 11 demonstrates another example of how these patterns or repetitive complex behaviors were classified as catatonia by Kahlbaum. It involved a 24-year-old male with alternating melancholy and mania in association with catatonia. He displayed stereotypical behavior in the form of repeatedly rubbing feces on his face. In addition, he displayed mannerisms in speech by speaking in theatrical voice, features, and postures.

“*A very prominent symptom at this time was a revolting dirtiness (e.g., consistently relieved himself next to the chamber pot and rubbed feces on his face), in complete contradiction with his overall behavior during his quiet periods)…. At times, he wished to feel maniacal excitation and then walked around in his room while he talked to himself in a loud voice. These attacks became more frequent, and the patient spoke vociferously with theatrical features, postures, and gesticulations…. During periods when he was less mysteriously silent or taciturn, he liked to speak to himself in a theatrical, declamatory manner or to play-act, and this pathetic mood gave him the idea that during his childhood, people told him that he should become an actor*.

Case 12 describes a young male, who presented with alternating melancholy, mania and catatonia. Kahlbaum described various non-contextual fixated peculiarities of activities, which represents mannerisms of breathing and walking.

“*The patient had a prominent tendency to strange, fixated positions, which occurred during the second stage of exhalations and the singular habits of walking with slow, regular, measured steps, combined with other similar striking voluntary movements*”.

Case 17 was a 33-year-old male who developed catatonia. Kahlbaum described various non-contextual fixated repetitive actions (stereotypies). He wrote:

“*During this period, he didn’t utter a sound, except for some soft groans at one point … Later, he was reported to have repeatedly tossed and turned in bed at night and gnashed his teeth*”.

Case 20 was a 40-year-old female who developed catatonia. Despite using the term stereotyped manner to describe her walking, the presentation emphasized a description of mannerism. In addition, she had several non-contextual activities (stereotypies) such as repetitive hand movements, repetitive walking around people, and turning. Kahlbaum reported:

“*At times, she walked around in a stereotyped manner, moved her hands in a circle before her breast, walked around other persons, turned around on one spot, or turned plates or pieces of meat around, and other similar turning movements*”.

**Table 1** summarizes Kahlbaum’s presentations of complex behaviors with the suggested assignment of these behavior patterns into either mannerisms or stereotypies according to the definitions in section 4.3.

**Table 1:** Summarizes Kahlbaum’s presentations of complex behaviors with the suggested assignment of these behavior patterns into either mannerisms or stereotypies using the definitions in section 4.3.

Case number	Description of the activity or behavioral content	Pathological process	Assignment into mannerisms vs. stereotypies
2^nd^ case	Peculiar habit of leaving some food, even if given minimal quantities	Non-contextual peculiar, fixated pattern	Mannerism
3^rd^ case	Fanatic compulsion with cold-water treatment	Non-contextual repeated and fixated activity	Stereotypy
3^rd^ case	Repeated, twisting a garment in her hands, twisting clothes into a sausage.	Non-contextual repeated and fixated activity	Stereotypy
4^th^ case	Rubbing all hair off her and having a compulsion to undress.	Non-contextual repeated and fixated activity	Stereotypy
6^th^ case	Repeated tendency to eat directly from the plate without using cutlery.	Non-contextual repeated and fixated activity	Stereotypy
11^th^ case	He consistently relieved himself next to the chamber pot and rubbed feces on his face.	Non-contextual repeated and fixated activity	Stereotypy
11^th^ case	At times, he wished to feel maniacal excitation and then walked around his room, talking to himself in a loud voice.	Non-contextual peculiar, fixated pattern	Mannerism
11^th^ case	These attacks became more frequent, and the patient spoke vociferously with theatrical features, postures, and gesticulations.	Non-contextual peculiar, fixated pattern	Mannerism
11^th^ case	He liked to speak to himself in a theatrical, declamatory manner or to play-act, and this pathetic mood gave him the idea that during his childhood, people told him that he should become an actor.	Non-contextual peculiar, fixated pattern	Mannerism
12^th^ case	The singular habit of walking with slow, regular, measured steps	Non-contextual repeated and fixated activity	Stereotypy
14^th^ case	Exaggerated politeness and	Non-contextual peculiar, fixated pattern	Mannerism
14^th^ case	Repeated kissing hands	Non-contextual repeated and fixated activity	Stereotypy
17^th^ case	He repeatedly tossed and turned in bed at night and gnashed his teeth.	Non-contextual repeated and fixated activity	Stereotypy
20^th^ case	She walked around in a stereotyped manner	Non-contextual peculiar, fixated pattern.	Mannerism
20^th^ case	She moved her hands in a circle before her breast, walked around other persons, turned around on one spot, turned around plates or pieces of meat, and did other similar movements.	Non-contextual repeated and fixated activity.	Stereotypy

### 3.2 Catatonic mannerisms and stereotypies post-Kahlbaum:

Kraepelin described stereotypies and mannerisms in the 8^th^ edition of his Dementia Praecox and Paraphrenia textbook among patients with primary psychotic illness (7). He defined stereotypies as expressive movements that may become progressively simplified and persist for years with the loss of any original idiosyncratic purpose. He alternatively described mannerisms as strikingly unnatural distortions of common activities, characterizing them as abnormal in content, whereas stereotypies were abnormal in quantity (7). Bleuler (1857-1939) followed Kraepelin’s conceptualization of stereotypies and mannerisms while reporting that both phenomena can occur in normal individuals (8). Kleist (1879-1960) focused on the qualitative abnormal psychomotor phenomena of catatonia as a basis for classification. Kleist described several qualitative behaviors that can be captured under current terms of mannerisms, such as “pseudo-expressive” movements, smiling facial expressions that are not congruent with the emotional state, and pseudo-reactive movements, such as shrugging the shoulders. Unlike Kraepelin, he did not use the term mannerisms but used the term stereotypies to describe both activities (9) interchangeably. Kleist also used the terms rituals, compulsions, and obsessions to describe some of these non-contextual, fixated, and repetitive patterns or activities. He described that the subject might be irritated if prevented from performing these activities. However, generally, there is no anxiety associated with these actions, which tends to differentiate this from what is seen in obsessive-compulsive disorder (10). Leonhard’s (1944) description of various classifications of catatonia focused on the simple motor aspects of stereotypies. It stated that patients who have a mixture of akinesia and hyperkinesia can present with a jerky manner of movements, where the natural grace of movement is absent due to the loss of the harmonious interplay of the individual motor acts, rendering them meaningless, non-reactive, and without expressive nature (11). He also explained that when these movements become aimless and occur uniformly, he labeled them stereotypy or iteration. He described the speech utterance of these patients as short and ungrammatical. In his description of manneristic catatonia, a form of systematic schizophrenia, he suggested that this form had fewer involuntary movements and that motor activities become more stereotyped. His description of mannerisms either took the form of commission highlighting the fixated manner of performing the activity or omission by the fixated refusal of food or toilet, which resembles the current use of negativism. Hence, Leonhard's description of stereotypies focused on motor repetition, while mannerisms included more behavioral activities and focused on the unique pattern of these activities, including various presentations of negativism (11). Jones (1965) described several examples of stereotypies in patients with schizophrenia. These examples were mainly in the simple motor forms such as repetitive movements of part or whole of a limb, rocking, repetition of a purposeful act, facial expression suggesting fear or embarrassment, or distaste, and contained mannerisms and stereotypies as described by Kraepelin (12).

Later authors attempted to clarify the overlap between mannerisms and stereotypies. Gelenberg (1976) characterized grimacing, stereotypies, and mannerisms as bizarre and repetitive behaviors and used these terms interchangeably (13). Edwards et al. suggested that mannerisms are a movement disorder that highlights unusual ways of performing a voluntary motor act, focusing on varied movements, which is generally consistent with Kraepelin’s work (14). This distinction implies a difference of intentionality or purpose between mannerisms and stereotypies, which is complicated by Kraepelin’s observation that stereotypies may at least initially hold idiosyncratic meaning to the patient.

Recent DSM editions provided inconsistent definitions and descriptions of stereotypies and mannerisms, focusing more on simple motor actions than complex behaviors. For example, both DSM-III and DSM-III-R described catatonia as a subtype of schizophrenia, noting stupor or mutism, negativism, catatonic rigidity, catatonic excitement, or catatonic posturing, and with no reference to mannerisms or stereotypies (15, 16).

DSM-IV continued to recognize catatonia as a sub-type of schizophrenia but included mannerisms and stereotypies in its diagnostic criteria (17). However, it combined both under the category of peculiarities of voluntary movements, as evidenced by posturing (voluntary assumption of inappropriate or bizarre postures), stereotyped movements, prominent mannerisms, or prominent grimacing. Both DSM-IV and DSM-IV-TR described stereotyped movements in the appendices as repetitive, seemingly driven, and nonfunctional motor behaviors, with all the examples reflecting simple motor movements or acts such as handshaking, waving, body rocking, and head banging (17, 18). This definition was not specific to catatonia, and its appendix does not define mannerisms. There was no definition of these phenomena in the broader context of catatonia. The Bush-Francis Catatonia Rating Scale and Taylor (1990) definition identified stereotypies as motor abnormalities in the frequency of non-goal-directed activity and were connected to verbigeration, while mannerisms were defined as odd, though purposeful, acts that were inherently abnormal (19, 20).

Both DSM-5 and DSM-5-TR have defined stereotypies in the context of catatonia as repetitive/abnormally frequent, non-goal-directed activities (3, 21). Meanwhile, mannerisms were described as odd, circumstantial caricatures of normal actions, and no examples are provided for either. However, the DSM-5 index described mannerisms as a peculiar and characteristic individual style of movements, actions, thoughts, or speech (21). This definition is broader than the one provided in the criteria for catatonia, all of which created confusion and ambiguity. This lack of clarity and precision in the definitions highlights the need for further refinement in the field. DSM-5-TR does not define mannerisms or stereotypies in the index (3).

### 3.3 Mannerisms and stereotypies in other DSM diagnoses

Both stereotypies and mannerisms have been long-described in neurodevelopmental disorders, including ASD. Recent DSM-IV, DSM-IV-TR, DSM-5, and DSM-5-TR provided examples of the core phenomena instead of processes to differentiate between them (3, 17, 18, 21).

Stereotypies were also described under another condition in DSM-III-R, initially named “stereotypy/habit-forming disorder” and renamed as “stereotypic movement disorder” in subsequent editions (16). It was defined as repetitive, seemingly driven, and nonfunctional motor behavior (e.g., hand shaking or waving, body rocking, head banging, mouthing of objects, self-biting, picking at skin or bodily orifices, hitting own body). Of interest is that this definition provided a more comprehensive, more precise, and easily understandable explanation of stereotypies as a behavioral phenomenon that resembles the description provided by Kahlbaum in his monograph (2). In addition, it offered a broader extension of the phenomena beyond simple motor movements to include instrumental behaviors, e.g., head banging. However, it did not guide the recognition and description of complex behavioral manifestations of stereotypies (2).

In both ASD and stereotypic movement disorder, catatonia was not recognized as an exclusion criterion when these symptoms were observed, despite catatonia being highly comorbid among patients with developmental disorders (22, 23).

The lack of consistency in the DSM series’ description of these phenomena is complicated by the increased recognition of catatonia in developmental disorders. Stereotypies in patients with developmental disorders, such as repeated self-injury and head banging, have been shown to respond successfully to treatments classically used for catatonia, e.g., lorazepam and ECT (23–25). This leads to a question about the common origin of these symptoms and whether catatonia might be poorly characterized, leading to under-recognition.

### 3.4 Repetitive movements/behaviors in neurology literature

Several authors utilized “repetitive behavior” as an umbrella term to refer to the broad and often disparate class of behaviors characterized by repetition, rigidity, invariance, and inappropriateness. While such a definition included various manifestations of stereotypies and mannerisms, they also included neurological and movement disorders such as dyskinesia and tics (26). Many of these disorders resemble movements observed in catatonia, and they might even respond to similar treatments, such as benzodiazepines. However, obtaining a good history that includes the onset of these movements or activities, their progress, course, association with other catatonia symptoms, other psychiatric or neurological disorders, and response to treatment provides a valuable tool for clinicians to aid in the differentiation and hence, appropriate recognition and treatment (27, 28).

### 3.5 Catatonia presentation as a complex behavior in recent psychiatric literature

While simple motor movements were recognized as stereotypies or mannerisms, emerging literature has increasingly recognized them in complex behaviors. McDaniel & Spiegel (2010) described in a case series the repetitive water intake to the point of hyponatremia in 4 patients, alongside other catatonic signs (5). Three patients had symptom resolution with standard treatment for catatonia. Another report by Laux et al. (2021) also described the resolution of polydipsia, with the other features of catatonia, in two manic patients with lorazepam and its immediate recurrence after discontinuing this agent (29).

Other authors have noted repetitive complex behaviors connected to the syndrome of catatonia in addition to polydipsia. Mahgoub et al. (2024) described a patient with bipolar disorder who was maintained on lithium and presented with polydipsia and polyuria (30). They highlighted that stereotypical drinking, which is a manifestation of catatonia, can occasionally be misdiagnosed as lithium-associated nephrogenic diabetes insipidus. Mormando et al. (2021) described a case of a patient with ASD and catatonia who presented with repetitive self-induced emesis (31). This patient had a 4-year history of unexplained emesis that had been unchanged despite interventions, including a craniectomy for a Chiari 1 malformation, behavioral interventions, antiemetics, and various medication trials. When the clinicians framed his emesis as catatonic stereotypy, a course of ECT resulted in a complete remission.

Varadarajulu & Mahgoub (2021) described a case of a young woman who presented with agitation, perseveration, “coprophagia,” eating feces, and drinking urine (4). She was initially diagnosed with schizophrenia with severe disorganization. Still, she did not improve with trials of three different antipsychotics (olanzapine, risperidone, aripiprazole), responding instead to a 1mg lorazepam daily within three days. In addition, Garman et al., 2024 described a case of a patient who presented with severe depression and catatonia, which manifested by excessive eating, staring, excitement, and mutism. The above symptoms, including excessive eating, were resolved with lorazepam. Their case emphasized how eating, as a complex behavior, can manifest catatonia, which can be recognized by comparing the presentation to the previous personal norm and recognizing the pattern of non-contextual repeated and fixated activity rather than focusing on the action itself (5). McDaniel & Spiegel (2010) also described ingesting metal objects and excess water as manifestations of complex stereotypical activities in catatonia (6). Assignment of such behavior as mannerism or a stereotypy significantly depends on the definition used. However, they notably co-occur, may be indistinguishable sometimes, and respond to treatments targeting catatonia.

These reports highlight the clinical benefits of broadening the concept of stereotypies and mannerisms.

**Table 2** summarizes various definitions of mannerisms and stereotypies in multiple editions of DSM-IV and DSM-5 (3, 17, 18, 21).

**Table 2:** Various descriptions of mannerisms and stereotypies in DSM-IV, DSM-IV-TR, DSM-5, and DSM-5-TR (3, 17, 18, 21).

DSM edition	Definition of mannerisms in catatonia	Definition of stereotypies in catatonia	Definition of mannerisms in ASD	Definition of stereotypies in ASD	Definition of stereotypical movement disorder	Definitions of mannerisms in the appendix	Definitions of stereotypies in the appendix
DSM-IV	There was no definition, and both were called peculiarities of voluntary movements.	There was no definition, and both were called peculiarities of voluntary movements.	No definition. They were used interchangeably with various examples provided.	No definition. They were used interchangeably with various examples provided	Repetitive, seemingly driven, and nonfunctional motor behavior	No definition	Repetitive, seemingly driven, and nonfunctional motor behaviors, with all the examples reflecting simple motor movements such as hand shaking, waving, body rocking, and head banging.
DSM-IV-TR	Same as DSM-IV	Same as DSM-IV	Same as DSM-IV	Same as DSM-IV	Same as DSM-IV	No definition	Same as DSM-IV
DSM-5	Odd, circumstantial caricatures of normal actions	Repetitive/abnormally frequent, non-goal-directed activities.	Same as DSM-IV	Same as DSM-IV	Same as DSM-IV	The peculiar and characteristic individual style of movements, actions, thoughts, or speech.	Same as DSM-IV
DSM-5-TR	Same as DSM-5	Same as DSM-5	Same as DSM-IV	Same as DSM-IV	Same as DSM-IV	No definition	No definition

## 4 Discussion

### 4.1 Mannerism and stereotypies: beyond simple motor movements

As common with observational monographs, Kahlbaum’s description of movements focused on their presence rather than their patterns. Their site and perceived utility lead them to be described as stereotypies, mannerisms, or even grimacing if they were facial. Kahlbaum described the complex behavioral manifestations of catatonia, as noted in his cases, and used stereotypy, stereotypical behaviors, idiosyncrasies, strange behaviors, and compulsions to describe the fixated peculiar or repetitive patterns. The literature gradually moved towards simple motor manifestations of catatonia until recently, when more complex presentations were again recognized. The challenges encountered in the recognition of the complex presentations of catatonia on a larger scale can be summarized in the following points:

### 4.2 The limited ability to characterize the pathology of behavioral processes as opposed to content

Descriptive psychopathology guides the analysis of thoughts and behaviors (32). Jaspers (1997) described behaviors utilizing the terms “form” and “content.” (33). He stated that “

*form must be kept distinct from content which may change from time to time, e.g., the fact that hallucination is distinguished from its content, whether this is a man or a tree, threatening figures or peaceful landscape. Perceptions, ideas, judgment, feelings, drives, and self-awareness are all forms of psychic phenomena; they denote the particular mode of existence in which content is presented to us*”.

From the above, we suggest that the analyses of movements or behaviors involve two levels: the action itself, or what can be referred to as (content), and the (process), or (form) of the movements or actions that reflect their underlying phenomena, such as excitation, inhibition, repetitive or unique pattern, and the context in which these movements or activities occur. We will primarily use the term process in this paper to avoid confusion.

Current DSM-5-TR and clinical scales presented examples (contents) of catatonia manifestation rather than their underlying common phenomena or processes (3). An example could be the description of grimacing, recognized as a separate catatonic sign, even though it likely represents manifestations of mannerisms and/or stereotypies localized to the facial muscles. As this conceptualization is applied to an example of common presentations of catatonia, like grimacing, the fact that a patient presents with a contorted facial expression, the recognition of the non-contextual fixated peculiar or fixated repetitive pattern of activities is more relevant than the nature of the action itself or its site, making the critical focus on the underlying pathological phenomena or (process) more appropriate.

### 4.3 Ambiguity, inconsistencies, the interchangeable use of both mannerisms and stereotypies, and the focus on simple motor movements

As we aim to provide a more detailed description of both mannerisms and stereotypies, which are manifestations of a process dysfunction, we can argue that in mannerisms, the impairment in the process is the tendency to have a ‘peculiar pattern’ in the absence of an appropriate context. On the other hand, the pathological process in stereotypies is likely related to the prolonged non-contextual fixated repetition of activities. The nature of these actions, their location, and their degree of complexity are less relevant than their peculiarity or repetition. This broader categorization helps us move beyond the limitations of describing these phenomena as simple movements or guessing their purpose.

Hence, we propose defining stereotypies as non-contextual, fixated repetition of activities while defining mannerisms as non-contextual, fixated peculiarities of activities (**Table 3**). It’s important to note that both mannerisms and stereotypies can co-occur, making the assignment of behaviors into either mannerisms or stereotypies less relevant for diagnosis or treatment than understanding their underlying pathological process.

**Table 3:** Mahgoub’s suggested definition of stereotypies and mannerisms.

Terms	Definition
Stereotypies	Non-contextual, fixated repetition of activities
Mannerisms	Non-contextual, fixated peculiarities of activities.

### 4.4 Norms and context references for complex behaviors

Identifying and measuring complex actions and behaviors is challenging, underscoring the field’s complexity and the need for more research (34). One of the main difficulties lies in focusing on content rather than process, as the latter requires a reference for normative behaviors (35). These norms can be personalized to an individual across their lifespan or based on other reference points such as operational, value-based, statistical, or typological norms. The value norm, for instance, takes the ideal as its concept of normality, such as the presence of clean white teeth for everyone. However, the absence of this ideal is only sometimes considered pathological and cannot be a common reference point. The DSM and many clinical scales use operationalism to generate normative references and categorize certain acts as pathological. This atheoretical approach is descriptive and requires predefined presentations and the satisfaction of chosen criteria. While operationalism can be practical, reliable, and easily generalizable for assessment, these standards dominate the DSM and clinical scales (delusions, hallucinations, disorganized behavior, etc.) (36).

In the context of catatonia, operationalism enables us to recognize simple motor movements or actions, such as grimacing, which is considered pathological based solely on its presence. However, this approach also creates a hurdle in the case of complex behaviors, as their assessment may need clinicians to identify changes in their patterns over time and the context of their occurrence. A patient’s individual norm thus becomes one of the essential factors for assessment.

The individual norm uses the level of consistent functioning that an individual maintains over time as a reference. As an example, excessive unwise spending is a common presentation seen as a state among patients with manic episodes, and it epitomizes excitement and poor impulse control. However, many people without manic episodes may struggle with poor financial planning, and in those cases, excessive spending might represent an enduring behavioral trait. If we solely use an operational frame of reference, many who habitually spend money in excess could be incorrectly perceived as symptomatic for a manic episode. Using the individual norm for reference underscores the importance of personalization in psychiatric diagnosis, requiring knowledge of previous levels of function and extended observation to recognize changes to the patterns or frequency of behaviors.

The context in which behaviors occur is a crucial dimension for a more accurate analysis of complex behaviors and may help further delineate psychopathology (36). This is evident in using context to differentiate agitation from aggression based on the patient’s interaction with people and the environment (37). The concept of context can also be used to assess features of catatonia, which is highlighted in the DSM-5-TR by examples of catatonic agitation and mutism. In catatonic agitation, the context is expected not to be related to external stimuli. With regards to mutism, aphasia was excluded from mutism, as the inability to form words is an expected finding in aphasia, and mutism is a lesser included part of the state norm of this condition (3).

Context, or its absence, appears to have particular relevance in assessing mannerisms. An example is case 14 of Kahlbaum, the patient with an excessive pattern of politeness. Polite speech and actions are normative based on the values in particular settings, such as healthcare. Excessive politeness that occurs in a fixated manner that appears out of context renders such complex behavior to be classified as a mannerism. In addition, elements of process and context were highlighted in Turner et al. (1999) review on repetitive movements and behaviors among patients with ASD and other developmental disorders, where she suggested that they are marked for being rigid, invariant, repetitive, and inappropriate (26).

Statistical norms measure individual deviations against average values, with extreme variations considered abnormal. Typological abnormalities are normative from a statistical, individual, and values-based perspective but still represent pathology compared to the general population. An example is the presence of goiter in females in some African tribes (38). Despite goiter potentially reflecting pathological iodine deficiency, it is considered a sign of beauty based on the view of the tribe’s “typological norm,” and the absence of goiter is considered abnormal. Both statistical and typological norms are less relevant for assessing complex behaviors.

### 4.5 Application of the phenomenological concept of mannerisms and stereotypies to complex behaviors

Applying this approach to the complex presentations of catatonia provided in the literature, including those provided by Kahlbaum or cases from recent literature, can help underscore its utility. An example of complex mannerisms from Kahlbaum’s case 3 is *“…she had a compulsion to alter the names of people around her; some of these were from her previous circle of acquaintances and home surroundings, and some were names she had invented”.* In this case, the alteration of the people’s names is as odd as the action itself (content); however, it is the (process) of applying this pattern to people with no understandable context that seems to occur in a repeated way, represents a clear manifestation of the pathology. This assessment of the action itself reflects a pathology based on operationalism. However, identifying a change in the pattern or frequency requires knowledge about the person’s previous consistent level of functioning and the process during that time to compare with the current pattern or process for identifying a pathological process, which is the non-contextual peculiar pattern in mannerisms or non-contextual repetitive or fixated patterns in stereotypies (**Table 1**).

Using the example of polydipsia from both Laux and McDaniel, we can see that drinking water is a routine normative activity. However, when the frequency of drinking water increases in the absence of appropriate contexts, such as thirst or dehydration, and in the presence of other associated features of catatonia, it can be categorized as stereotypical behavior. This excessive and uncontrolled drinking can have a significant impact on homeostasis and result in serious medical comorbidity, a condition described in the literature as “psychogenic polydipsia.” The focus here is mainly on the action of drinking rather than the process of non-contextual, repeated, fixated activity patterns. The standard suggested treatment is mainly fluid restriction, a logical but not definitive treatment to the underlying catatonia, and results in further morbidity and mortality. These examples underscore the importance of understanding the individual norm and the fluctuation over time to recognize various manifestations of process pathology in complex behavior.

Kahlbaum and Kraepelin described verbigeration associated with stereotypies, highlighting the significant overlap and similarities. If we apply our analysis to verbigeration, the quality of words (content) may be appropriate or impaired. In addition, it might be in the form of a single word or a more complex sentence. However, it is the change in the process that can be assessed over time and about individual norms that repetition of these words or complex sentences without appropriate context, whether these words are meaningful or meaningless, display the pathological process in stereotypies. Thus, an argument can be made that verbigeration is phenomenologically similar to stereotypies as a manifestation of a process pathology involving the non-contextual repetitive pattern that manifests in speech instead of through other movements. Additionally, thought perseveration is also described as a manifestation of catatonia, which can be argued to share the same phenomenon that manifests in the cognitive domain, and the content of the thought is less relevant, regardless of its accuracy or complexity.

## 5 Limitations

While our suggested definitions and framework for recognizing complex presentations of mannerisms and stereotypies can be helpful for clinicians, this approach might be challenging to incorporate into various assessment tools as it does not utilize operationalism for normative reference.

## 6 Conclusion

Mannerisms and stereotypies are well-recognized manifestations of catatonia. Several definitions have been proposed and sometimes used interchangeably, leading to ambiguity and inconsistency in the DSM and psychiatric literature. The historical focus on differentiating mannerisms and stereotypies from each other has provided little added value to the recognition and treatment. Examples of simple motor movements have dominated catatonia descriptions, and the focus on underlying phenomena has been limited. Kahlbaum’s monograph provided several examples of mannerisms and stereotypies as complex behaviors without providing a systematic process to recognize and describe them. We suggest that evaluating complex behaviors in their appropriate context, including the patient’s personal norms of behavior, and linking them to the underlying pathological processes can provide clinicians with better ways to recognize complex or unique behavioral presentations of catatonia. As mannerisms and stereotypies might coexist during catatonia episodes, the identification of psychopathological processes associated with non-contextual peculiar or repetitive patterns of actions may aid recognition and treatment over simply limiting ourselves to assigning symptoms to a category.
